# Dual-Mode Flexible Sensor Based on PVDF/MXene Nanosheet/Reduced Graphene Oxide Composites for Electronic Skin

**DOI:** 10.3390/nano13010102

**Published:** 2022-12-25

**Authors:** Hu Liang, Libing Zhang, Ting Wu, Haijun Song, Chengli Tang

**Affiliations:** 1 School of Mechanical Engineering and Automation, Zhejiang Sci-Tech University, Hangzhou 310018, China; 2 College of Information Science and Engineering, Jiaxing University, Jiaxing 314001, China

**Keywords:** dual-mode, flexible sensor, electronic skin, human motion monitoring, PVDF/MXene Nanosheet/rGO

## Abstract

MXene materials have the metallic conductivity of transition metal carbides. Among them, Ti_3_C_2_T_X_ with an accordion structure has great application prospects in the field of wearable devices. However, flexible wearable electronic devices face the problem of single function in practical application. Therefore, it is particularly important to study a flexible sensor with multiple functions for electronic skin. In this work, the near-field electrohydrodynamic printing (NFEP) method was proposed to prepare the composite thin film with a micro/nanofiber structure on the flexible substrate using a solution of poly(vinylidene fluoride)/MXene nanosheet/reduced graphene oxide (PMR) nanocomposites as the printing solution. A dual-mode flexible sensor for electronic skin based on the PMR nanocomposite thin film was fabricated. The flexible sensor had the detection capability of the piezoresistive mode and the piezoelectric mode. In the piezoresistive mode, the sensitivity was 29.27 kPa^−1^ and the response/recovery time was 36/55 ms. In the piezoelectric mode, the sensitivity was 8.84 kPa^−1^ and the response time was 18.2 ms. Under the synergy of the dual modes, functions that cannot be achieved by a single mode sensor can be accomplished. In the process of detecting the pressure or deformation of the object, more information is obtained, which broadens the application range of the flexible sensor. The experimental results show that the dual-mode flexible sensor has great potential in human motion monitoring and wearable electronic device applications.

## 1. Introduction

With the development of science and technology, the demand for flexible wearable electronic devices is gradually increasing, among which a flexible electronic skin has attracted extensive attention [1,2,3]. Flexible electronic skin usually has good convenience and sensing ability, which can capture the motion parameters of the human body and convert them into electrical signals [4]. Flexible electronic skin has gradually been widely used in the fields of medical care, human–computer interaction, motion detection, health monitoring, software robots, and other fields [5,6].

The flexible pressure/strain sensor is usually one of the important components of a flexible electronic skin. According to the detection principle, a flexible pressure/strain sensor can be divided into the piezoresistive type [7,8,9,10,11,12], capacitive type [13,14], piezoelectric type [15,16,17], and frictional type [18]. Different types of flexible pressure/strain sensors are suitable for different types of pressure/strain responses according to their principles. Among them, the piezoelectric pressure/strain sensor is suitable for the detection of transient pressure/strain signals. Roy et al. reported a piezoelectric pressure sensor based on polyvinylidene fluoride (PVDF)/graphene oxide (GO) composites [19]. The fabricated sensor could quickly detect the pressure of 10 Pa, and it has a good application prospect in detecting human physiological signals. Yang et al. fabricated a flexible piezoelectric pressure sensor based on poly dopamine modified BaTiO_3_/PVDF composite film [20]. The fabricated sensor could improve the dynamic response capability by preparing an inorganic/organic composite piezoelectric material film. Luo et al. demonstrated the flexible piezoelectric sensor based on a PVDF/SWCNT composite film for electronic skin [21]. The fabricated sensor had high sensitivity and fast response capability. However, piezoelectric pressure/strain sensors cannot detect the pressure on an object in the steady state, which limits its application in the field of electronic skin [22]. Tian et al. reported a resistive pressure sensor prepared by laser inscribed flexible graphene [23]. The fabricated sensor had a large pressure sensing range and extremely high sensitivity. Qin et al. prepared a piezoresistive pressure sensor with high sensitivity and high reliability based on MXene/PVB [24]. The prepared sensor could detect the subtle bending and releasing activities of the human body. Yang et al. used a composite material of rGO and MXene as a pressure sensitive material, and used a polyester filament (PET) as the fiber matrix to fabricate a flexible pressure sensor [25]. The fabricated sensor has many advantages such as good linearity, fast response speed, and stable performance, and it has great application potential in the detection of human joint motion and respiration. Ma et al. combined the large surface area of rGO and the conductivity of MXene to prepare an aerogel pressure sensor with an MXene/rGO composite 3D structure [26]. The sensor showed better sensing performance than rGO or MXene with a single component, and it could clearly test human pulse signals. This indicates that MXene, rGO, and other two-dimensional materials have good application prospects in the design of electronic skin.

Traditional piezoresistive pressure sensors usually demonstrate narrow monitoring ranges (<10%) and lower sensing sensitivity at small strains [27]. Piezoresistive sensors can detect the pressure/strain state and deformation state of the object, but it is difficult to detect the bending strain direction of the object because it is insensitive to the loading speed and bending speed of the strain. In addition, piezoresistive pressure sensors have obvious shortcomings in the detection of multi-mode pressure signals, which also limits its application range. Due to the properties of piezoelectric materials, piezoelectric sensors have a good ability to detect transient pressure/strain changes. However, piezoelectric sensors find it difficult to detect the steady-state pressure/strain signal of the object. Therefore, the single-mode pressure/strain sensor finds it difficult to detect different forms of the pressure/strain signals. The practical application of a flexible pressure/strain sensor for electronic skin is complicated, and it needs to sense transient and steady-state pressure/strain signals [28]. Therefore, in order to improve the sensing ability of a flexible pressure/strain sensor for electronic skin, it is necessary to fabricate a multifunctional flexible sensor that can detect multiple pressure/strain information at the same time. This is beneficial to improve the performance of a flexible sensor for electronic skin.

The thin film process of the sensitive material used for the flexible pressure/strain sensor has an important influence on its performance. Physical preparation methods of flexible sensors include screen printing [29,30], electrospinning [31,32], photolithography [33,34], spin coating [35,36], inkjet printing [37,38], and so on. Gong et al. studied the formation mechanism of sponge and designed a flexible strain sensor based on a 3D electrospun carbonized sponge [39]. The sensor has good fast response speed and stability, which can monitor the motion of the wrist joint, elbow joint, and finger in real-time. Yi et al. fabricated functional circuits on 3D freeform surfaces via intense pulsed light-induced zinc mass transfer [40]. However, different methods have certain problems in the preparation process. Photolithography requires expensive equipment and complex preparative processes, which increases the preparation costs. The printing accuracy of traditional inkjet printing is poor, and it is difficult to prepare high-precision patterns with micro/nano structures. At the same time, ink drying may cause some problems such as the coffee ring effect and nozzle clogging, which affects the consistency of morphology and the printing stability. In the process of preparing the flexible sensor by electrospinning, due to the large distance between the nozzle and the substrate, the spinning jet is prone to flutter, which makes it difficult to achieve high-precision patterning. The near-field electrohydrodynamic printing (NFEP) technology is a new micro/nano-scale printing technology based on electric field force pulling ink to prepare micro/nano structures. By shortening the distance between the nozzle and the substrate, it avoids the whip of the printing jet. Through the motion of the substrate, a pattern or thin film with micro/nano structures can be prepared. Therefore, the method can be used to fabricate a flexible pressure/strain sensor [41,42,43,44].

In this work, the mixed solution of PVDF/MXene nanosheet/reduced graphene oxide (PMR) nanocomposites was directly printed on the flexible substrate to fabricate a thin film with a micro/nano fiber structure by the NFEP method, which effectively improved the performance of the flexible sensor. A dual-mode flexible sensor for electronic skin, which could sense transient pressure/strain and steady-state pressure/strain signals, was fabricated using the PMR nanocomposite thin film as a sensitive sensing unit. The surface morphology and microstructure were characterized in detail using scanning electron microscopy (SEM) and optical microscopy. The piezoresistive signal and piezoelectric signal were detected for the PMR dual-mode flexible sensor, and the dual-mode sensing ability of the flexible sensor was tested. In addition, the PMR dual-mode flexible sensor was also used to detect information of wrist motion and other parts of the human body.

## 2. Materials and Method

### 2.1. Materials

A Ti_3_C_2_T_X_ multilayer nanosheet was purchased from Jilin Yiyi Technology Co. Ltd., China. PVDF (Molecular weight 370,000) was purchased from Arkema, France. rGO (outer diameter 0.5~5 μm, >99 wt%, thickness 0.35 nm) was purchased from Hangzhou Hangdan Optoelectronics Technology Co. Ltd., Hangzhou, China. Acetone (≥99.5%) was purchased from Hangzhou Qingchen Chemical Co. Ltd., Hangzhou, China. N,N-dimethylformamide (DMF, ≥99.5%) and anhydrous ethanol (analytical pure) were purchased from Jiangsu Qiangsheng Functional Chemical Co. Ltd., Suzhou, China. Acetone was purchased from the Linan Qingshan Chemical Reagent Factory. Polydimethylsiloxane (PDMS, Model BD film KRR-20) was purchased from Hangzhou Balde New Material Technology Co. Ltd., Hangzhou, China.

### 2.2. Preparation of PMR Mixed Printing Solution

**Figure 1:**
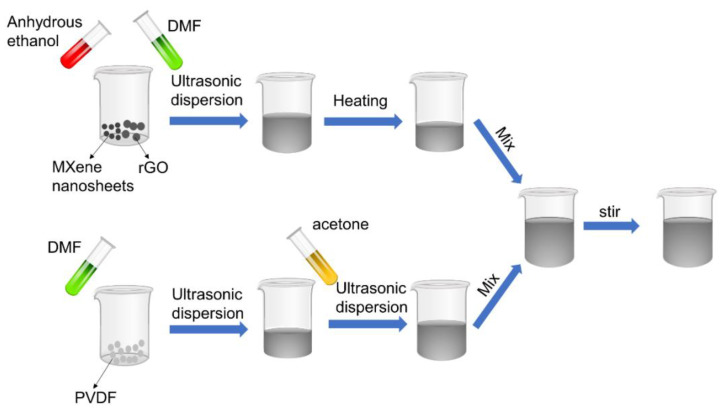
The preparation process of the PMR nanocomposite printing solution.

The preparation process of the PMR mixed printing solution is shown in Figure 1. Aa total of 0.5 g of the multi-layer MXene nanosheet and 0.03 g of rGO powder were put into a beaker, respectively. A sample of 3 mL of DMF and 2 mL of anhydrous ethanol were added into the beaker for ultrasonic treatment for 2 h. The mixed solution was dried in a drying oven at 50 °C for removal, which was prepared into the MXene/rGO mixed solution. Then, 1.5 g of PVDF powder was added to 4 mL DMF solution and sonicated for 1 h, and then 1.5 mL acetone was added and sonicated for 20 min to form the PVDF solution. Finally, the MXene/rGO mixed solution was poured into the PVDF solution and magnetically stirred for 12 h to obtain the PMR nanocomposite printing solution (PVDF/MXene-rGO = 3:1). Using the same method, other PMR mixed solutions were prepared at different mass ratios (PVDF/MXene-rGO) of 1:1, 2:1, 4:1, and 5:1, respectively.

### 2.3. Preparation of PMR Nanocomposite Thin Films

**Figure 2:**
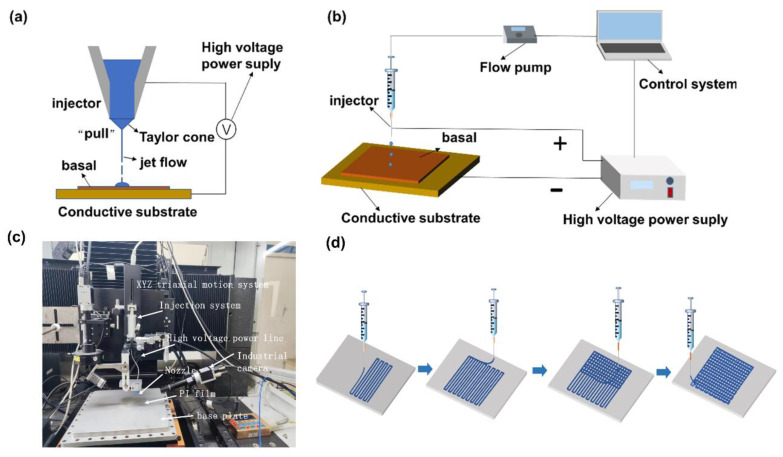
The NFEP process of the PMR nanocomposite thin film. (**a**) The printing principle of the NFEP technology. (**b**) The composition of the self-developed NFEP equipment. (**c**) Physical picture of the NFEP equipment. (**d**) Printing path of the PMR nanocomposite thin film.

The PMR nanocomposite thin film was mainly prepared by the NFEP method. The NFEP method is a non-contact direct forming technology, which can generate a jet from the top of a conical meniscus under the action of an electric field between the nozzle and the substrate. Unlike traditional inkjet printing technologies such as thermal inkjet printing or piezoelectric inkjet printing, the printing principle of the NFEP technology is shown in Figure 2a. The NFEP technology is a pull processing technology that pulls ink and generates the jet diameter with a micro/nano scale. The composition of the self-developed NFEP equipment is shown in Figure 2b,c. The self-developed NFEP equipment mainly consists of a three-axis motion platform, a control system, an injection system, a high-voltage power supplier, and a visual monitoring system. The three-axis platform consists of the X axis, Y axis, and Z axis. The X axis and Y axis are composed of the linear motor and the grating ruler, and the Z axis is composed of the AC servo motor and the grating ruler. The control system is composed of hardware and software. The hardware is composed of an industrial computer and motion controller, and the software is composed of a management system, motion control system, and power control system. The motion control system controls the motion of the platform, and the power control system realizes the control and processing of the high-voltage power supplier. The injection system includes the injection pump, syringe, stainless steel nozzle, and other parts. The visual monitoring system is composed of the CCD camera, the gigabit network card, the light source, and the brightness controller, which can monitor the jet shape and pattern of the printing process in real-time. The control system controls the voltage of the high-voltage power supplier, the flow of the injection system, and the motion of the three-axis motion platform, which realizes the preparation of the thin film with micro/nano fiber structures using the PMR nanocomposite printing solution. The printing path of the PMR nanocomposite thin film with micro/nano structure fibers is shown in Figure 2d. The PMR nanocomposite solution was used as the printing solution. The self-developed NFEP equipment was used to prepare the PMR nanocomposite thin film on the polyimide (PI) flexible substrate with a stainless steel nozzle of the inner diameter of 0.41 mm and the outer diameter of 0.71 mm. Through the motion program code, the motion platform was controlled to move back and forth in the X- and Y-directions to form the pattern of a network structure. Finally, the PMR nanocomposite thin film composed of micro/nano fibers was prepared on the PI flexible substrate. The process parameters were the applied voltage of 3 kV, the flow rate of 1.5 uL/min, the printing height of 0.2 mm, and moving velocity of 5 mm/s. The prepared PMR nanocomposite thin films were placed into a drying oven for drying.

### 2.4. Fabrication of PMR Dual-Mode Flexible Sensor

**Figure 3:**
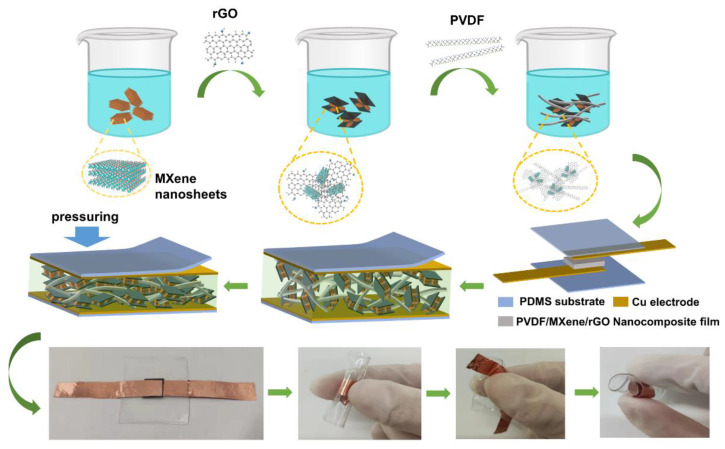
Schematic diagram of the PMR dual-mode flexible sensor.

The PMR dual-mode flexible sensor used for the electronic skin was composed of two copper electrodes, two PDMS films, and one sensitive material thin film, as shown in Figure 3. The middle layer was the sensitive unit of the PMR nanocomposite thin film with a thickness of about 82 µm, which was wrapped with electrodes on both sides. Two copper electrodes were encapsulated with two flexible PDMS films. The size of the PMR dual-mode flexible sensor was 4.0 cm × 3.3 cm, and the effective working area was 1.2 cm × 1.2 cm. Finally, the fabricated the PMR dual-mode flexible sensor was placed in a container to prevent pollution and corrosion.

### 2.5. Characterization and Measurement

The morphology and structure of the PMR nanocomposite thin films were characterized using scanning electron microscopy (SEM, MAGELLAN-400, USA). The crystal phase structures of the PMR nanocomposite thin films were characterized by X-ray diffraction (XRD, Haoyuan DX-2700BH, China). The response performance of the flexible sensor was measured with a digital source meter (Keithley 6510 Source Meter SMU Instrument, Beaverton, OR, USA). The thickness of the PMR nanocomposite thin film was measured by means of the step meter (DektakXT, Boyue Instruments Co. Ltd., Shanghai, China). The Fourier transform infrared spectrum (FTIR, Thermo Scientific Nicolet 460, Woodland, CA, USA) was performed to analyze the FTIR spectra of the thin films.

The change rate of the sensor increased with the increase in the applied external pressure, which can clearly distinguish different pressures. The change rate of resistance can be expressed as follows:

(1) $$Y=\frac{\Delta R}{R}=\frac{R-R_{p}}{R}$$

where *Y* is the change rate of resistance; *R_p_* is the resistance of the flexible sensor under the action of pressure; and *R* is the resistance of the sensor under no pressure.

## 3. Results and Discussions

### 3.1. Sensing Mechanism of the PMR Dual-Mode Flexible Sensor

**Figure 4:**
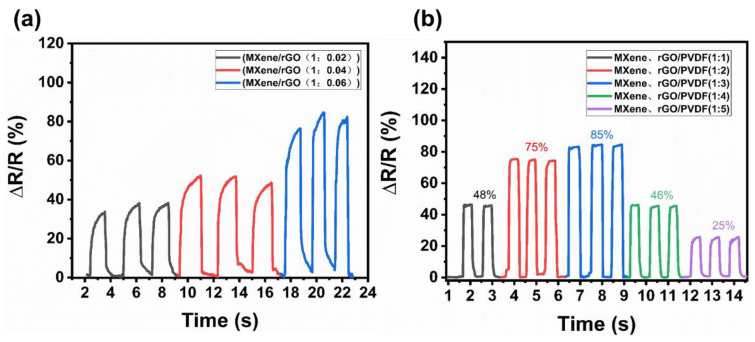
(**a**) Resistance change rates of the MXene/rGO nanocomposite thin films with different ratios (1:0.02, 1:0.04, and 1:0.06, respectively). (**b**) Resistance change rates of MXene-rGO/PVDF nanocomposite thin films with different ratios (1:1, 1:2, 1:3, 1:4, and 1:5, respectively).

To obtain the optimal mixing material ratio, the MXene/rGO mixed solutions with different mass ratios were prepared in the comparative experiment, and the mass ratios of MXene and rGO were 1:0.02, 1:0.04, and 1:0.06, respectively. The MXene/rGO mixed solutions with different mass ratios were coated on the interdigital electrodes to make simple flexible sensors. By applying the same pressure (2 kPa), the pressure tests were performed on the composite with different MXene/rGO mass ratios. As shown in Figure 4a, the resistance change rates of composites with an MXene/rGO ratio of 1:0.02, 1:0.04, and 1:0.06 were about 38%, 53%, and 82%, respectively. When the mass ratio of MXene/rGO was 1:006, the resistance change rate reached the maximum. This indicates that the composite material with this proportion has dd sensitive resistance response and good sensing performance under the same pressure conditions. However, as the proportion of rGO continued to increase, the fabricated flexible sensor became fragile due to the too small density of rGO, and it was difficult to form a continuous macrostructure. Therefore, the mass ratio of the MXene/rGO mixed solutions was selected as 1:0.06. In further experiments, mixed solutions of MXene/rGO and PVDF with different mass ratios were prepared, and the mass ratios were 1:1, 1:2, 1:3, 1:4 and 1:5, respectively. Using these five mixed solutions as printing solutions, the PMR nanocomposite thin films were prepared by the NFEP equipment. In the comparative experiments, the relative resistance test was carried out on the PMR nanocomposite thin films of the same size prepared with different mass ratios without a pressure signal. The initial resistance of the pressure sensors with ratios of 1:1 to 1:5 was 0.82 kΩ, 17.5 kΩ, 220 kΩ, 900 MΩ, and 2350 MΩ, respectively. If the initial resistance was too small, the change of resistance was not obvious when the PMR nanocomposite thin film deformed under the action of the pressure signal. If the initial resistance was too large, it could show insulation characteristics. Therefore, the resistance of the flexible sensor with a ratio of 1:3 is more reasonable. Furthermore, the same pressure (2 kPa) tests were performed on the composite with different MXene-rGO/PVDF mass ratios (1:1 to 1:5). As shown in Figure 4b, the resistance change rates were 48%, 75%, 85%, 46%, and 25%, respectively. With the increase in PVDF, the resistance change rate of the composite material increased first and then decreased. When the mass ratio of MXene-rGO/PVDF was 1:3, the change rate of resistance was the highest. According to the above experimental conclusions, the PMR nanocomposite thin film with a mass ratio of MXene-rGO/PVDF (1:3) was used as the sensitive unit of the PMR dual-mode flexible sensor.

The sensitive material of the PMR dual-mode flexible sensor was mainly composed of PVDF, the MXene nanosheet, and rGO. The surface of rGO had a large number of functional groups such as carboxyl, hydroxyl, and epoxy, which makes it easy to combine with other substances. rGO is very sensitive to sensing pressure and strain. At the same time, rGO has a larger sheet diameter, so the larger sheet size of rGO is conducive to the construction of the conductive network for the flexible sensor. MXene is a new type of two-dimensional (2D) material, which is mainly composed of transition metal carbides, nitrides, or carbonitrides. Due to the large number of active groups on the surface of the MXene material, the MXene material has the metal conductivity of transition metal carbides. Among them, Ti3C2TX, with an accordion structure, has great application prospects in the field of wearable devices. In addition, according to the principle of similar phase dissolution, the hydroxyl groups on the surface of the MXene nanosheet can be well mixed with those on the surface of rGO [26]. The MXene material has excellent electrical conductivity and mechanical flexibility, which fully meets the requirements of a flexible sensor for material conductivity and flexibility. At the same time, the electrical conductivity of MXene and the larger sheet size of rGO can synergistically improve the piezoresistive performance of the flexible sensor. However, the aspect ratio of the MXene nanosheet is relatively small, which makes it difficult to form a continuous MXene nanosheet macrostructure [45]. On one hand, the added PVDF can combine the hybrid structure composed of MXene and rGO to form a continuous macrostructure, which can make the PMR composites form an integral elastomer and improve the stability of the sensitive material [46]. In addition, PVDF is a partially crystalline polymer. The crystal forms of PVDF crystallization can be divided into the α-phase, β-phase, γ-phase, and δ-phase, among which the β-phase is the most polar. Due to the presence of the β-phase, PVDF exhibits stronger piezoelectric properties than other polymers. On the other hand, from the perspective of piezoelectric properties, the added rGO and MXene nanosheet could improve the flexibility, thermal stability, and electrical conductivity of PVDF. At the same time, rGO can promote the transition from the α-phase of non-polar crystals to the β-phase of polar crystals, which improves the dielectric constant and piezoelectric coefficient of the nanocomposites [47]. Meanwhile, the piezoelectric β-phase of PVDF has spontaneous polarization, which can be reversed under the action of an electric field. Due to the high-voltage electric field generated by the high voltage applied in the NFEP method, the PVDF can be polarized, so that the molecular dipoles with random orientation distribution are aligned along the direction of the electric field. Therefore, the macroscopic piezoelectric effect can be generated [48].

The hybrid structure of the MXene nanosheet and rGO had good electron transport properties. PVDF is a high molecular polymer with insulating properties. In the composites, MXene nanosheet and rGO were intertwined with each other in PVDF to form electronic channels. The addition of the MXene nanosheet/rGO improved the carrier transport efficiency, which makes the PMR composites have a good piezoresistive effect. In addition, PVDF with piezoelectric properties was the main material in the composite material, so the PMR nanocomposites still had a higher resistance. PVDF inhibits the conduction of carriers in the composite material to a certain extent, which prevents the composite material from becoming a conductor material. Therefore, the piezoelectric properties of PVDF can be exerted in the detection of piezoelectric signals, so that the PMR composites have a piezoelectric effect. The combination of piezoresistive and piezoelectric materials provides a basis for detecting the pressure or strain signals of the PMR dual-mode flexible sensor. When detecting piezoresistive signals, under the action of the external pressure/strain signal, the number of contact points between the conductive networks formed by the rGO and MXene material increases, which leads to a decrease in the resistance of the sensitive material. When detecting piezoelectric signals, the sensitive material is impacted or released by the instantaneous dynamic pressure/strain, which results in rapid deformation, and the PMR flexible sensor generates an electrical signal under the action of the piezoelectric effect [49].

**Figure 5:**
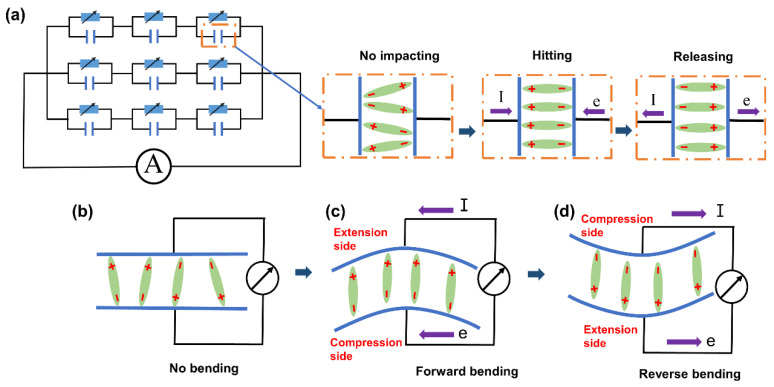
(**a**) Schematic diagram of the sensor being subjected to applied pressure and released pressure. (**b**) Unbent state. (**c**) Forward bending state. (**d**) Reverse bending state.

The schematic diagram of the PMR dual-mode flexible sensor is shown in Figure 5a. The sensitive unit of the PMR dual-mode flexible sensor is equivalent to being composed of several piezoresistive materials and piezoelectric materials in parallel. When the PMR dual-mode flexible sensor is not stimulated by the pressure/strain signal, the resistance of the flexible sensor remains unchanged, and the electric dipole oscillates randomly within a certain range without any piezoelectric output. When the PMR dual-mode flexible sensor is stimulated by the external pressure/strain signal, the resistance value of the piezoresistive material decreases due to its deformation. At the same time, due to the deformation of the piezoelectric material, both ends of the piezoelectric material output an instantaneous positive current. When the applied pressure/strain is removed, the resistance value of the piezoresistive material increases. In addition, the deformation of the piezoelectric material is gradually restored, and both ends of the piezoelectric material output an instantaneous negative current. In addition, during the bending process of the flexible sensor, the bending of the PMR dual-mode sensor can generate lateral strain and reduce the polarization along the polarization direction. As shown in Figure 5b, the flexible sensor was not bent, the electric dipole oscillated randomly within a certain range, and there was no piezoelectric output. A negative piezoelectric potential occurred on the compressed side of the sensor, while a positive piezoelectric potential appeared on the elongation side. In addition, a flow of electrons from the compression side to the elongation side was generated to balance the piezoelectric potential. As shown in Figure 5c,d, different bending states of the flexible sensor had different elongation and compressed sides.

### 3.2. Characterization of the PMR Dual-Mode Flexible Sensor

**Figure 6:**
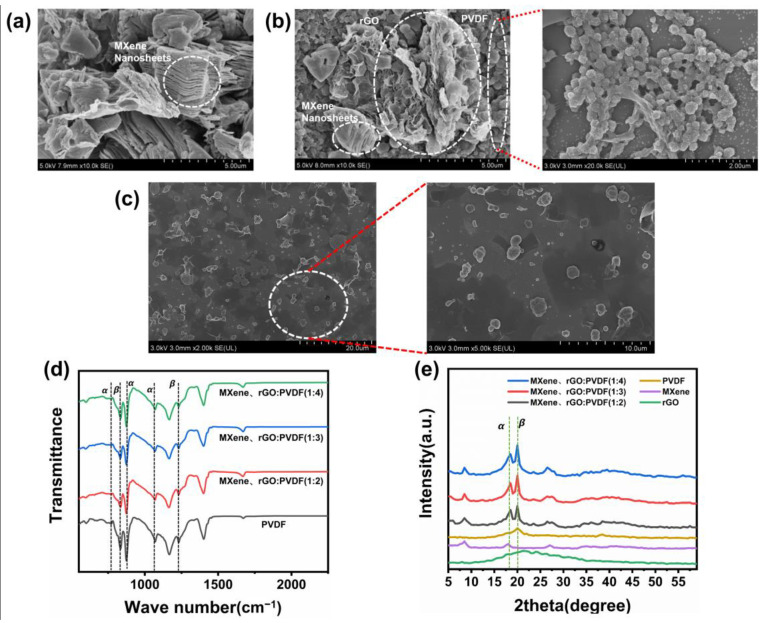
Characterization of the nanocomposite thin films. (**a**) SEM image of the MXene/rGO nanocomposite thin film surface. (**b**) SEM image of the PMR nanocomposite thin film surface. (**c**) Low magnification SEM image of the PMR nanocomposite thin film. (**d**) FTIR spectra of the PVDF, PVDF/MXene/rGO nanocomposite thin films. (**e**) XRD pattern of the PVDF, MXene, rGO, and PVDF/MXene/rGO nanocomposite thin films.

By adding rGO with a large surface area into the MXene nanosheet with metal conductivity, the three-dimensional (3D) hybrid structure composed of two materials could form rich 3D spatial networks. As shows in Figure 6a, the MXene nanosheet in the MXene/rGO nanocomposite thin film had a multilayer structure. However, due to the relatively small density of the rGO material, when a small amount of rGO was added to the MXene material, it was difficult to form a stable macrostructure with the hybrid material, which led to poor durability and stability of the prepared MXene/rGO nanocomposite thin film. Adding PVDF to the MXene/rGO nanocomposites could solve this problem. The SEM image of the PMR nanocomposite thin film is shown in Figure 6b,c, and the experimental results showed that the hybrid structure composed of multilayer MXene and flake rGO was homogeneously mixed in PVDF. PVDF is a high molecular polymer with good flexibility. PVDF can be used as a binder in the mixed solution to crosslink the MXene material with rGO material to form a stable macrostructure. The FTIR spectrum is shown in Figure 6d, where for the β-phase, the peaks in the infrared spectrum were located at 834 cm^−1^ and 1230 cm^−1^, and the infrared absorption band characteristics of the α-phase were located at 763 cm^−1^, 875 cm^−1^, and 1079 cm^−1^ [50]. When rGO was added to PVDF, the characteristic bands of the α and β phases were stronger than those without rGO, indicating that the content of the α and β phases increased. The increase in the β-phase is beneficial to improving the piezoelectric properties of the PMR nanocomposite thin film. Therefore, the nanofillers added to PVDF can interrupt the ordered crystal symmetry of PVDF and form the electroactive β-phase [47]. The XRD pattern of PVDF, MXene, and rGO with different ratios of PMR composites is shown in Figure 6e. The prominent peak of MXene in the XRD pattern was located at 2θ equal to 8.9°, which denotes the MXene (002) plane diffraction. The XRD pattern of rGO had protrusions at 2θ equal to 22°, and PVDF had (200) and (110) diffractions of the β-phase at 2θ equal to 21° in the XRD pattern. Compared with the diffraction peaks of pure PVDF, PMR had better piezoelectric properties.

### 3.3. Sensing Properties of the PMR Dual-Mode Flexible Sensor

**Figure 7:**
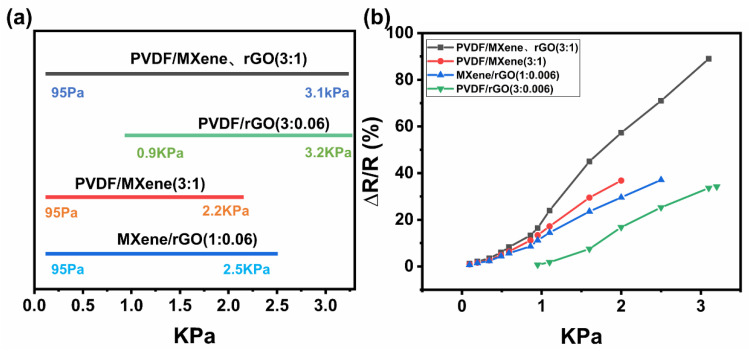
(**a**) Pressure detection range of different nanocomposite thin films and (**b**) the change in the relative resistance of different nanocomposite thin films.

The PVDF/rGO (mass ratio 3:0.06) nanocomposite thin film, PVDF/MXene (mass ratio 3:1) nanocomposite thin film, and MXene/rGO (mass ratio 1:0.06) nanocomposite thin film were prepared by the NFEP method. As shown in Figure 7a, the sensing properties of different types of nanocomposite thin films were investigated. Through comparison, the results showed that the PVDF/rGO composite thin film found it difficult to effectively detect a pressure less than 0.9 kPa. This was due to the sheet-diameter structure of rGO and its relatively small content. Therefore, when the external pressure was small, there were fewer connections between the conductive networks. The sensing range of the PVDF/MXene composite thin film was relatively small. Because the accordion-structured MXene had metallic conductivity, MXene with the accordion structure had a strong ability to sense the tiniest pressure in the composite material. When the pressure was greater than 2.2 kPa, the resistance change rate did not change with the increase in pressure. MXene with metallic conductivity and the larger diameter rGO interacted synergistically in the PVDF/MXene/rGO composite thin film. Therefore, the PVDF/MXene/rGO nanocomposite thin film had a larger detection range. In addition, the changes of relative resistance of different nanocomposite thin films were compared. As shown in Figure 7b, the changes in the relative resistance of the PVDF/MXene/rGO composite thin film was the largest under the same pressure.

**Figure 8:**
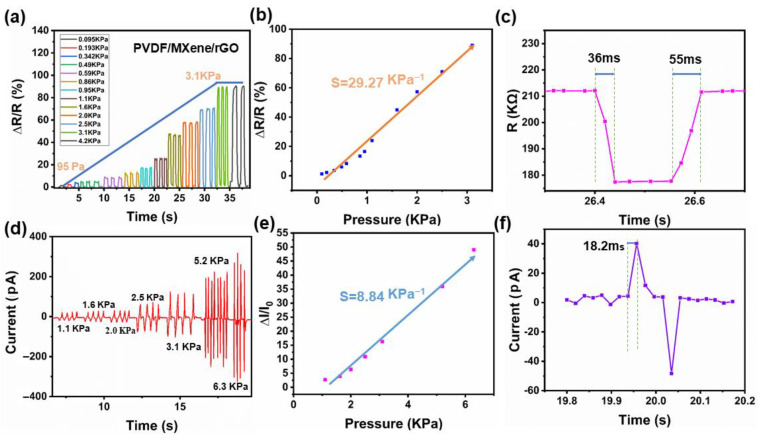
(**a**) Sensing range of the piezoresistive properties of the PMR dual-mode flexible sensor. (**b**) Sensitivity of the piezoresistive mode. (**c**) Response/recovery time of the piezoresistive mode. (**d**) Sensing range of the piezoelectric mode. (**e**) Sensitivity of the piezoelectric mode. (**f**) Response time of the piezoelectric mode.

Piezoresistive sensing characteristics of the PMR nanocomposite films are shown in Figure 8a–c. Figure 8a shows the effective sensing range of the piezoresistive mode. The minimum sensing limit of the piezoresistive mode was 95 Pa, and the maximum sensing limit was 3.1 kPa. Once the pressure exceeded the maximum sensing limit pressure, the resistance change rate remained unchanged. The change rate of resistance under external force from 3.1 kPa to 4.2 kPa is shown in Figure 8a. As shown in Figure 8b, the sensitivity of the piezoresistive mode was 29.27 kPa^−1^. As shown in Figure 8c, the response/recovery time of the piezoresistive mode was 36/55 ms by applying and releasing the pressure of 0.85 kPa. The piezoelectric sensing properties of the PMR nanocomposite thin film are shown in Figure 8d–f. As shown in Figure 8d, the minimum response limit of the piezoelectric mode was 1.1 kPa, and the maximum response limit was 6.3 kPa. Once the pressure signal was greater than 6.3 kPa, the piezoelectric signal appeared in the unstable measurement state, so the effective sensing range under the piezoelectric mode was 1.1 kPa–6.3 kPa. As shown in Figure 8e, the sensitivity of the piezoelectric mode was 8.84 kPa^−1^. As shown in Figure 8f, the response time of the piezoelectric mode was 18.2 ms by applying and releasing a pressure of 2.2 kPa. In terms of the sensing range, the piezoresistive mode could effectively detect small pressure signals and the piezoelectric mode was able to detect larger pressure signals. These two modes complement each other with a larger detection range.

This work was compared with a recently reported wearable pressure sensor [26,28,45,51,52,53,54,55,56]. As shown in Table 1, compared with the studies in these studies, the fabricated dual-mode flexible sensor had higher sensitivity and faster response speed. Therefore, the dual-mode sensor could sense both the transient signal and the steady-state signal, which is conducive to reducing the fabricating cost of the flexible sensor.

### 3.4. Practical Applications of PMR Dual-Mode Flexible Sensor

**Figure 9:**
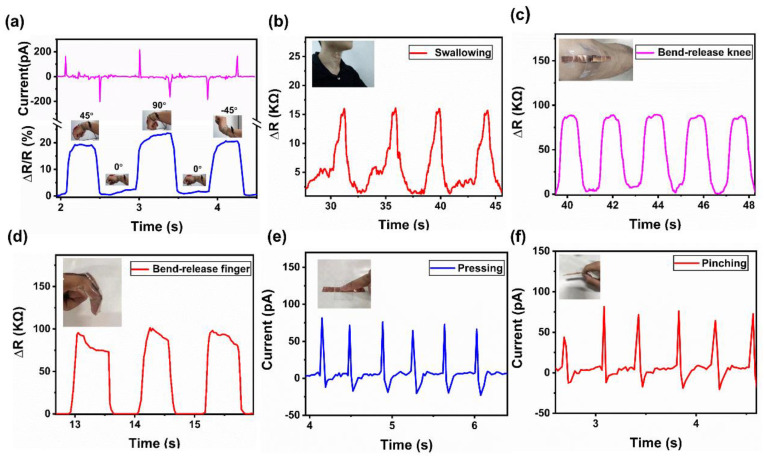
Practical applications of the PMR dual-mode flexible sensor for electronic skin. (**a**) Sensing response of the flexible sensor for detecting wrist bending and release. (**b**) Sensing response of the flexible sensor for swallowing action. (**c**) Sensing response of the flexible sensor for bending and straightening of the knee. (**d**) Sensing response of the flexible sensor for bending and releasing with a finger. (**e**) Piezoelectric signal with quick finger press. (**f**) Piezoelectric signal with finger rapid pinching.

In order to test the application of the PMR dual-mode flexible sensor for electronic skin, it was applied to different parts of the human body to detect human motion signals. As shown in Figure 9a, the PMR dual-mode flexible sensor was fixed on the wrist, and the bending and release of the wrist caused the change in resistance. When the wrist was bent down by 45° and 90°, respectively, an instantaneous positive piezoelectric signal was generated, and the resistance decreased, but the relative resistance change increased. The instantaneous peak value of the piezoelectric signal can reflect the wrist bending speed, and the relative resistance change can reflect the wrist bending angle. When the wrist returned to its initial state, an instantaneous negative piezoelectric signal was generated, and the resistance increased, but the relative resistance change decreased. When the wrist was bent upward by 45°, a negative piezoelectric signal was generated, and the resistance decreased, but the relative resistance change increased. Therefore, the PMR dual-mode flexible sensor could detect various motion signals of the wrist including the bending angle, bending direction, and bending speed. As shown in Figure 9b, the PMR dual-mode flexible sensor was installed on the throat of the tester. The flexible sensor could detect the changes in the throat muscles during swallowing. As shown in Figure 9c, the bending and straightening of the knee caused the regular change in the resistance signal of the flexible sensor. As shown in Figure 9d, the flexible sensor was fixed on the index finger. When the index finger was bent, the flexible sensor was bent and deformed, and the resistance of the sensor changed. It could clearly monitor the bending state of the index finger and the relative resistance change increased with the increase in the bending angle. When the index finger was straightened, the pressure/strain was released and the resistance value of the flexible sensor became the initial resistance. As shown in Figure 9e,f, by pressing and pinching the sensor with the fingers quickly, the sensor could sense and output the piezoelectric signal. When pressure was applied, the sensor outputted an instantaneous positive piezoelectric signal. When the pressure was released, the sensor outputted an instantaneous negative piezoelectric signal. Therefore, the output piezoelectric signal in piezoelectric mode can help to detect more instantaneous pressure signals of the human body. Therefore, the piezoelectric signal and piezoresistive signal output by the sensor can identify a variety of human motion information, which has good application prospects in the field of human motion detection.

## 4. Conclusions

In conclusion, based on the properties of the PVDF/MXene/rGO nanocomposites, the PMR dual-mode flexible sensor was fabricated by using the NFEP method. Due to the unique sensing principle of the PMR dual-mode flexible sensor, it had a dual-mode detection ability with the piezoresistive mode and the piezoelectric mode. In the piezoresistive mode, the sensitivity was 29.27 kPa^−1^, and the response/recovery time was 36/55 ms. In the piezoelectric mode, the sensitivity was 8.84 kPa^−1^, and the response time was 18.2 ms. Under the cooperation of the two modes, the two modes can complement each other in the sensing pressure range. In addition, the PMR dual-mode flexible sensor can detect a variety of information about the pressure/strain signal such as the bending angle, bending direction, and bending speed, which makes up for the shortcomings that the piezoresistive flexible sensor cannot sense the direction and speed of the bending signal. The experimental results show that the PMR dual-mode flexible sensor has great potential in human motion monitoring and wearable electronic devices.

## Figures and Tables

**Table 1:** Performance comparison of the PMR flexible sensor.

Mode	Materials	Sensitivity	Responsiveness	Reference
Piezoresistive	PVDF/MXene/rGO	29.27 kPa^−1^	36/55 ms	This work
	MXene/rGO	22.56 kPa^−1^	245/212 msk.	[26]
	SF@MXene Ti_3_C_2_T_x_	25.5 kPa^−1^	40/35 ms	[45]
	MXene/PVDF-TrFE	0.51 kPa^−1^	150/150 ms	[51]
	MXene/bacterial	12.5 kPa^−1^	167/121 ms	[52]
	P(VDF-TrFE)/rGO	14.5 kPa^−1^	-----------	[28]
Piezoelectricity	PVDF/MXene/rGO	8.84 kPa^−1^	18.2 ms	This work
	PVDF nanorod	5.17 kPa^−1^	150 ms	[53]
	PET-Self-Powered	0.018 kPa^−1^	60 ms	[54]
	Graphene/P(VDF-TrFE)	0.76 kPa^−1^	<100 ms	[55]
	AuNW	>1.14 kPa^−1^	<17 ms	[56]
	P(VDF-TrFE)/rGO	1.62 V/kPa	-----------	[28]

## Data Availability

Not applicable.
